# A Distinct Translation Initiation Mechanism Generates Cryptic Peptides for Immune Surveillance

**DOI:** 10.1371/journal.pone.0003460

**Published:** 2008-10-21

**Authors:** Shelley R. Starck, Yongkai Ow, Vivian Jiang, Maria Tokuyama, Mark Rivera, Xin Qi, Richard W. Roberts, Nilabh Shastri

**Affiliations:** 1 Division of Immunology and Pathogenesis, Department of Molecular and Cell Biology, University of California, Berkeley, California, United States of America; 2 Howard Hughes Medical Institute and Department of Biochemistry, University of Texas Southwestern Medical Center, Dallas, Texas, United States of America; 3 Department of Chemistry, Chemical Engineering, and Biology, University of Southern California, Los Angeles, California, United States of America; Institut Pasteur, France

## Abstract

MHC class I molecules present a comprehensive mixture of peptides on the cell surface for immune surveillance. The peptides represent the intracellular protein milieu produced by translation of endogenous mRNAs. Unexpectedly, the peptides are encoded not only in conventional AUG initiated translational reading frames but also in alternative cryptic reading frames. Here, we analyzed how ribosomes recognize and use cryptic initiation codons in the mRNA. We find that translation initiation complexes assemble at non-AUG codons but differ from canonical AUG initiation in response to specific inhibitors acting within the peptidyl transferase and decoding centers of the ribosome. Thus, cryptic translation at non-AUG start codons can utilize a distinct initiation mechanism which could be differentially regulated to provide peptides for immune surveillance.

## Introduction

The presentation of peptides, derived from endogenously synthesized proteins, by the major histocompatibility complex class I molecules (MHC I) is essential for immune surveillance by the CD8^+^ T cell repertoire [1], [2], [3]. The peptides are produced by the antigen processing pathway which begins with proteasomal degradation of newly synthesized proteins and ends with presentation of pMHC I on the cell surface [4], [5], [6]. Interestingly, the peptide mixture contains proteolytic products of not only conventional AUG initiated open-reading frames (ORFs) but also those encoded by alternative reading frames (ARFs) with or without AUG initiation codons called cryptic translation products or cryptic pMHC I [1]. Although cryptic pMHC I are expressed at low levels, they are nevertheless capable of eliciting CD8^+^ T cell responses specific for a variety of tumors, virus infected or even normal cells (reviewed in [1], [7]).

Previously, we had used T cell assays to detect cryptic pMHC I on the cell surface and in cell extracts [8], [9], [10]. These measurements showed that not only non-AUG initiation codons, such as CUG, could be used to translate antigenic peptides, but that the CUG codon was decoded with a leucine residue. Initiating translation with a leucine, rather than the canonical methionine was very unusual. Established models of translation suggest that initiation at non-AUG start codons is mediated by the methionine charged initiator tRNA (Met-tRNA_i_^Met^) through ‘wobble’ interactions with the anticodon [11], [12]. Accordingly, the non-AUG initiation codon, CUG should have been decoded as a methionine residue suggesting the existence of unusual translation mechanisms for generating cryptic pMHC I.

The display of pMHC I on the cell surface is a key mechanism for immune surveillance of infected cells synthesizing new viral proteins [4]. Interestingly, viruses have evolved alternate mechanisms to subvert normal translational controls [13]. For example, many viral gene products are translated using internal ribosome entry sites (IRES) [14]. The IRES allows direct binding of ribosomal initiation complexes to appropriate start codons without the requirement for 5′ to 3′ scanning. On the other extreme, some insect viruses do not require any known initiation factors [15], [16]. The downstream capsid protein coding sequence of the Cricket Paralysis (CrPV) or the *Plautia stali* intestine viruses are translated by initiation at the non-AUG codons GCU or CAA using alanine or glutamine residues respectively. Remarkably, the IRES elements of these insect viruses can also function in mammalian cells suggesting that they interact with highly conserved features of the eukaryotic ribosome. Thus, it was possible that IRES-like mechanisms could have been used for translating cryptic pMHC I.

Internal ribosome entry was apparently unnecessary for translation because expression of cryptic pMHC I was inhibited by insertion of upstream hairpin sequences [10]. Furthermore, unlike presentation of the AUG-initiated peptide, which was inhibited by upstream out-of-frame AUG codons, the presentation of the CUG-initiated peptide was inhibited by upstream CUG rather than AUG codons. This observation suggested that the ribosomes initiating translation at CUG codons were actually scanning for CUG codons and thus differed from conventional ribosomes that scan for AUG initiation codons. In addition, sodium arsenite, an inhibitor of methionine initiation [17], affected presentation of an AUG-initiated peptide, but not a CUG peptide, suggesting the existence of a methionine-independent mechanism for eukaryotic ribosomes initiating at non-AUG start codons. Independently, a distinct set of ribosomes, termed the “immunoribosome”, has been proposed to generate peptides for presentation by MHC I [18]. Thus, protein synthesis may not only be linked to generation of pMHC I, but could involve novel translational mechanisms.

Here, we analyzed the ribosomal initiation complexes that recognize the initiation codon in mRNAs encoding a cryptic antigenic peptide. We show that non-AUG codons, such as CUG, engage ribosomes during the initiation step of translation. Moreover, these complexes can be distinguished from those that recognize conventional AUG codons by small molecule inhibitors that affect the P site of the ribosome/tRNA initiation complex.

## Results

### Ribosome initiation complexes recognize a non-AUG start codon

To characterize the molecular mechanism which permits non-AUG start codons, such as CUG to engage ribosomes, we used the primer extension inhibition assay called toeprinting [19] (**Fig. S1**). The ribosomes are allowed to assemble on a mature mRNA during the translation initiation step but are prevented from translating the entire message by the elongation inhibitors cycloheximide and sparsomycin. The location of the ribosomes bound to the mRNA is then determined by extending a [^32^P]-labeled complementary 3′ primer with reverse transcriptase (RT) and the RT products are visualized after fractionating by gel-electrophoresis. The size of the RT products, measured at a single nucleotide resolution by comparison with sequencing reactions run on the same gel, indicates the distance traversed by RT on the mRNA.

**Figure 1:**
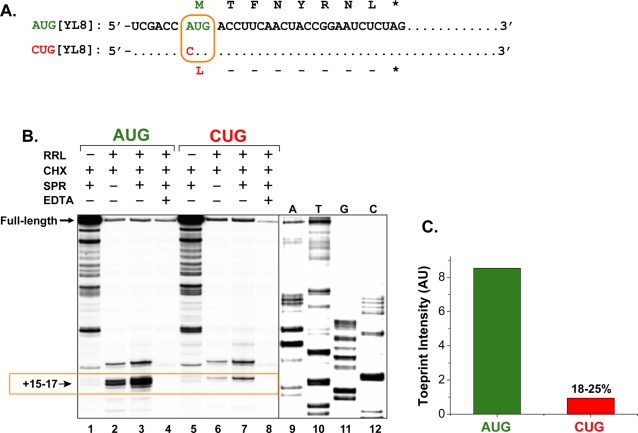
Ribosome initiation complexes recognize cryptic CUG start codons. (A) The mRNA sequences of the AUG[YL8] and CUG[YL8] constructs used for toeprinting differ at a single nucleotide in the initiation codon (boxed). The amino acid sequence encoded by AUG[YL8] (MTFNYRNL, MYL8) or the CUG[YL8] (LYL8) are shown above and below the nucleotide sequences respectively. (B) Toeprinting analysis on AUG[YL8] and CUG[YL8] mRNAs using rabbit reticulocyte lysate as the ribosome source (see methods for experimental details). A band at +15–17 nucleotides downstream of the A of the AUG codon (boxed) is the toeprint and represents the leading edge of ribosomal initiation complexes at either the AUG or the cryptic CUG start codons. The bands above the toeprint bands (∼12 nt from the start codon) are non-specific because they were unaffected by translation initiation inhibitors. Sequencing lanes shown are for the CUG[YL8] mRNA. The data shown are representative from 5 independent toeprinting experiments. (C) Toeprint intensity of the AUG and CUG bands from (B) is shown in arbitrary Phosphoimager units (AU). On average, the CUG toeprint represents 18–25% of the intensity of the AUG toeprint.

We synthesized mRNAs from the same cDNA constructs used earlier to generate pMHC I in transfected cells (**Fig. 1A**). Cells translating the LYL8 (LTFNYRNL) peptide derived from the *H60* histocompatibility gene, or its analog MYL8 (MTFNYRNL), present the peptide-K^b^ MHC I complex on the cell surface [8], [9]. This gene has been used as a model to study cryptic translation because the AUG or the CUG initiation codons can be decoded as Met or Leu residues to yield Met-YL8 (AUG[YL8]) or the Leu-YL8 (CUG[YL8]) peptides (**Fig. 1A**). When the AUG[YL8] mRNA was used as a template in the absence of ribosomes, a strong band representing the full-length cDNA fragment as well as many smaller fragments were detected (**Fig. 1B, lane 1**). The smaller fragments are likely due to secondary structures in the mRNA template which inhibit the progress of RT at the lower 30°C temperature used in this assay. In the presence of rabbit reticulocyte lysate (RRL), used as a source of ribosomes, and the elongation inhibitor cycloheximide, the intensity of the full-length fragment markedly decreased and new bands appeared (**lane 2**). The size of these RT products was determined by comparison with the sequencing reactions (**lanes 9–12**) as +15–17 nucleotides downstream of the AUG codon where the first nucleotide of the AUG triplet is +1. Another larger band terminating at +12 nucleotides downstream of AUG was also reproducibly observed which is likely to be non-specific because it was unaffected by translation initiation inhibitors (see below). The intensity of these bands was further increased when sparsomycin, another elongation inhibitor, was also added to the reaction (**compare lanes 2 and 3**). These bands represent ribosomes bound to the mRNA because they were not detected when EDTA was added to disrupt ribosomes by chelating Mg^2+^ ions (**lane 4**). We conclude that ribosomal initiation complexes can be observed at the conventional AUG codon by toeprinting. Furthermore, these initiation complexes are likely to include the small 40S ribosomal subunit which contains initiator Met-tRNA_i_^Met^ to provide specificity for the AUG initiation codon. Furthermore, because both cycloheximide and sparsomycin inhibit elongation by binding to the 60S ribosomal subunit indicates that these complexes also contain the large 60S ribosomal subunit.

The CUG[YL8] mRNA showed a remarkably similar pattern of bands compared to the AUG[YL8] mRNA (**Fig. 1B, lanes 5–8**). A +15 nucleotide toeprint band was detected in the presence of cycloheximide alone (**lane 6**) and its intensity was enhanced when sparsomycin was also added to the reaction (**lane 7**). Again EDTA inhibited the CUG initiation complexes confirming that the toeprint required intact ribosomes (**lane 8**). Notably, in multiple experiments, the CUG toeprint was reproducibly weaker than the AUG toeprint representing ∼18–25% of the intensity of the AUG toeprint (**Fig. 1C**).

The location of the toeprint, 15–17 nucleotides downstream of the initiation codon, indicates that the first aminoacyl-tRNA, usually Met-tRNA_i_^Met^, is placed in the P site of the ribosome in contrast to the A site which is the first point of entry for all other aminoacyl-tRNAs [19]. We conclude that ribosomal initiation complexes can be assembled at the cryptic CUG initiation codons at the same location as the canonical AUG initiation codons with the tRNA positioned in the P site of the ribosome.

### Ribosomal binding to the CUG codon occurs during initiation

**Figure 2:**
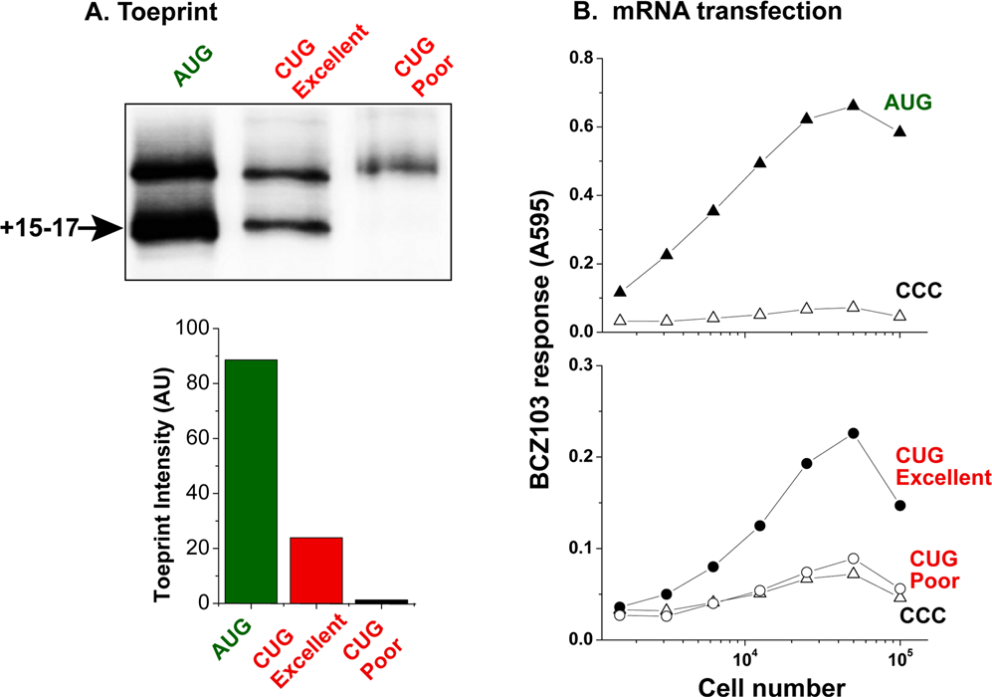
Initiation at the CUG codon depends upon the Kozak context *in vitro* and *in vivo*. (A) Toeprinting with AUG[YL8] and CUG[YL8] mRNAs in either an “Excellent Kozak” (UCGACC[CUG]A) or a “Poor Kozak” context (GCGUCCCUGA). The toeprints at +15–17 nucleotides downstream of the AUG or CUG start codons are indicated. The bar graph below shows the intensity of toeprints in arbitrary Phosphoimager units (AU). The data shown are representative of three independent toeprinting experiments. (B) The mRNAs used for toeprinting in (A) and an mRNA with the CCC initiation codon as a negative control, were transfected into K^b^-L cells. Three hours later the presentation of peptide-K^b^ complexes on the cell surface was measured using the LYL8-K^b^ or MYL8-K^b^ specific BCZ103 hybridoma. The β-galactosidase activity induced in the activated T cell hybridoma was measured using the substrate chlorophenol red-β-D-galactopyranoside, which yields a colored product with an absorbance at 595 nm.

Ribosomes recognize mRNA codons during the initiation as well as the elongation steps of protein synthesis [12], [20]. However, only the initiation step is strongly influenced by the nucleotides surrounding the initiation codon [21]. To assess whether the ribosomal binding to the CUG codon was due to the initiation step, we carried out toeprint analysis with mRNAs containing CUG codons with varying nucleotide contexts. We used mRNA containing the AUG initiation codon as a positive control and compared it with mRNAs containing a CUG codon in an “Excellent” (UCGACC**CUG**A) versus a “Poor” context (GCGUCC**CUG**A). These nucleotide sequences were previously identified in a screen for optimal initiation context for the CUG initiation codon [10]. Compared to the strong toeprint band at +15–17 nucleotides with the AUG codon and a weaker band with the CUG codon in an “Excellent Kozak” context, a toeprint band was not detected when the CUG was in a “Poor Kozak” context (**Fig. 2A**, CUG Poor). These changes in the intensity of the CUG toeprint by the Kozak context indicate that the CUG codon is involved in the initiation step.

To further establish the impact of Kozak context on CUG initiation, we transfected the same mRNAs into L-cell fibroblasts expressing the K^b^ MHC I. Peptide presentation on the cell surface was measured using the BCZ103 hybridoma, which is specific for K^b^ bound LYL8 or the MYL8 peptides [8]. Robust T cell activation was observed with the AUG[YL8] mRNA but not with the CCC[YL8] mRNA showing that the AUG initiation codon in the same “Kozak” context as CUG was efficiently used in translation (**Fig. 2B**, top panel). In contrast, the BCZ103 hybridoma responded to cells transfected with the CUG[YL8] mRNA, but only when the CUG was in an “Excellent Kozak” context (**Fig. 2B**, lower panel). Changing the CUG context to a “Poor Kozak” context diminished the level of antigen presentation to almost that of the CCC[YL8] background. Note that measurement of peptide presentation with cells transfected with mRNA instead of cDNA constructs also rules out potential variables such as transcription, splicing and mRNA export. We conclude that differences in toeprint intensity *in vitro* as well as the amount of translated products produced in living cells are consistent with recognition of the CUG codon in the decoding center of the ribosomal P site during initiation.

Next, we characterized ribosomal initiation at the CUG codon for the key mediators that determine the specificity of this step; the small and the large ribosomal subunits and the initiator tRNA.

### CUG recognition requires 5′ cap and GTP hydrolysis but is independent of Met-tRNA_i_^Met^

**Figure 3:**
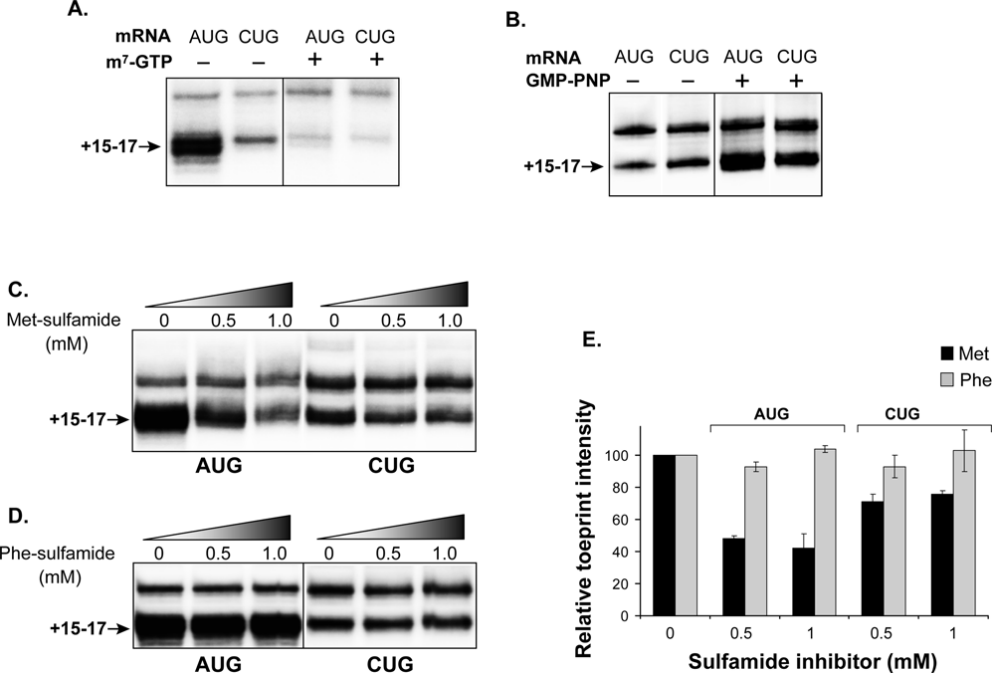
CUG recognition requires 5′-cap and GTP hydrolysis but is independent of Met-tRNA_i_^Met^. (A) Toeprint analysis of AUG[YL8] and CUG[YL8] mRNAs in the presence of translation initiation inhibitors cycloheximide and sparsomycin and the 5′-cap m^7^GTP analog (1 mM). (B) Toeprint analysis in the presence of the non-hydrolyzable GTP analog, GMP-PNP (0.4 mM). Cycloheximide and sparsomycin were not included in this experiment because GMP-PNP inhibits large ribosomal subunit assembly on the pre-initiation complexes. (C) Methionine-sulfamide (Met-sulfamide) inhibits toeprints in a dose-dependent manner on AUG[YL8] mRNA, but not CUG[YL8] mRNA. Met-sulfamide (along with cycloheximide and sparsomycin) were added during the 5 min preincubation prior to adding mRNA. (D) Phenylalanine-sulfamide (Phe-sulfamide) does not alter toeprints on either AUG or CUG mRNAs. Phe-sulfamide was added to toeprint reactions as carried out for Met-sulfamide described above. (E) Relative toeprint intensity is % of untreated sample from (C) with data from three independent experiments and from (D) with data from two independent experiments (mean+/−standard error).

Prior to scanning for the initiation codon, conventional, but not IRES-mediated, translation requires that ribosomes and initiation factors first bind to the 5′ m^7^G cap structure that is present in all eukaryotic mRNAs [12]. To assess directly whether CUG bound ribosomes required binding to the 5′ cap, we carried out toeprint analysis in the presence of m^7^GTP cap analog which competes for binding to the cap binding protein, eIF4E. Upon addition of mRNA, toeprints at both the AUG and CUG start codons were strongly inhibited (**Fig. 3A**), indicating that ribosomes recognizing the CUG as well as AUG initiation codons utilize the cap structure at the 5′ end of mRNAs. Taken together with the ability of stable hairpins to inhibit pMHC I presentation efficiency shown in functional assays [10], this result shows that initiation at CUG start codons, unlike IRES mediated initiation, requires linear scanning of the 5′ mRNA sequence beginning at the 5′ cap.

Next, we assessed whether GTP hydrolysis promoted the assembly of complete ribosomal initiation complexes at the CUG codon. In the absence of GTP hydrolysis, the 60S ribosomal subunit does not assemble on 48S pre-initiation complexes which contain the small 40S ribosomal subunit, several initiation factors and Met-tRNA_i_^Met^ bound at the AUG start codon [12]. These 48S pre-initiation complexes lacking the 60S subunit accumulate and produce toeprints like those obtained from the complete initiation complexes with both the small and large ribosomal subunits [22]. When the non-hydrolyzable GTP analog, GMP-PNP, was included in the toeprinting reactions, we observed an accumulation of pre-initiation complexes at both AUG and CUG start codons (**Fig. 3B**). Thus, initiation at the CUG codon is similar to the AUG codon in its requirement for GTP-hydrolysis and is consistent with assembly of a pre-initiation complex.

The formation of a 48S pre-initiation complex in turn requires the binding of 40S small ribosomal subunits loaded with initiation factors and Met-tRNA_i_^Met^. Loading of Met-tRNA_i_^Met^ onto 40S subunits requires methionyl-tRNA synthetase to provide a dedicated pool of Met-tRNA_i_^Met^ for AUG initiation [23]. Inhibitors of methionyl-tRNA synthetase block initiation at AUG start codons with Met-tRNA_i_^Met^ because they inhibit aminoacylation of tRNA with methionine [24]. We used methionine sulfamide (Met-sulfamide), a synthetic methionyl-tRNA synthetase inhibitor in the AUG versus CUG toeprint assay. Initiation on the AUG[YL8] mRNA, as seen by the toeprint at +15–17 nt, was inhibited in a dose-dependent manner (**Fig. 3C,E**). In contrast, the toeprint on CUG[YL8] mRNA was relatively resistant to Met-sulfamide (**Fig. 3C,E**). As a negative control, toeprints at both AUG and CUG mRNAs were unaffected in the presence of phenylalanine-sulfamide (Phe-sulfamide), a phenylalanyl-tRNA synthetase inhibitor (**Fig. 3D**). Thus, a portion of CUG-specific initiation complexes can assemble in the absence of Met-tRNA_i_^Met^ suggesting that a different aminoacyl-tRNA is present in the ribosomal P site. This result is in complete agreement with previous findings that CUG can be decoded with a leucine residue [8], [9], [10].

### Edeine inhibits AUG but not CUG initiation

**Figure 4:**
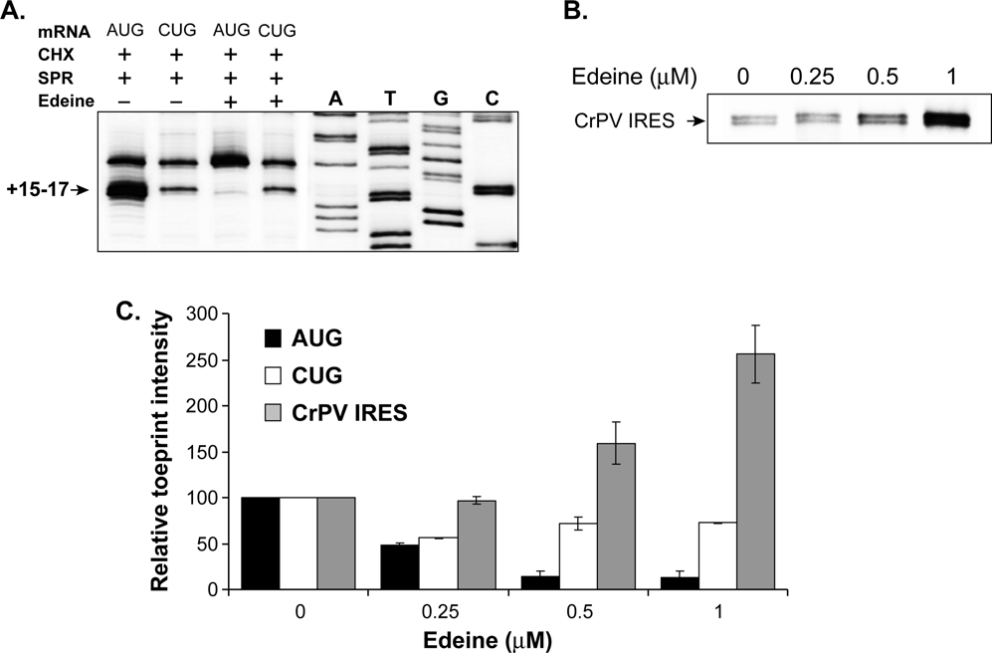
Edeine inhibits AUG but not CUG toeprints. (A) Toeprints of mRNAs with the indicated initiation codons in the absence or presence of edeine (2 µM). The toeprints at +15–17 nucleotides downstream of the AUG or CUG start codons are boxed. Data are representative of five independent experiments. Sequencing lanes shown are for the CUG[YL8] mRNA. (B) Edeine enhances the toeprints on the cricket paralysis virus (CrPV) IRES mRNA in a dose-dependent manner. (C) Relative toeprint intensity (% of untreated sample) of the AUG[YL8], CUG[YL8], and CrPV IRES mRNAs in the presence of indicated doses of edeine (mean+/−standard error). The intensity of the toeprint in the absence of edeine is set at 100%. Data are from two independent experiments.

Methionine-independent initiation at the CUG codon suggested that it may be possible to further distinguish the recognition of non-AUG versus AUG initiation codons with protein synthesis inhibitors. Edeine is a peptide antibiotic which inhibits translation in all organisms because it binds to the small 40S ribosomal subunit and disrupts the proper placement of initiator Met-tRNA_i_^Met^ within the P site of the ribosome (**Fig. S1**) [25]. Notably, translation via the CrPV IRES is resistant to edeine, consistent with Met-tRNA_i_^Met^-independent initiation [15], [26]. To assess potential differences in ribosomal recognition of AUG versus the non-AUG initiation codon, CUG, we carried out toeprint analysis in the presence of edeine. As expected, the AUG-specific toeprint was almost completely inhibited in the presence of edeine (**Fig. 4A**). In contrast, edeine had little effect on the CUG toeprint. To further confirm the differential effect of edeine on CUG initiation, we tested a range of edeine concentrations in the toeprint analysis and also included the CrPV mRNA as an edeine-resistant control (**Fig. 4B**). Again, in contrast to the toeprints at the AUG codon, the intensity of toeprints at the CrPV IRES as well as the CUG mRNA was not reduced but was even enhanced with increasing edeine concentration (**Fig. 4B,C**). We chose to compare CrPV IRES to CUG initiation since CrPV initiation complexes are known to be resistant to edeine, a universal inhibitor of translation initiation. These results are consistent with the notion that initiation events at CUG were mediated by an alternate initiation mechanism that did not utilize Met-tRNA_i_^Met^ in the P site of the ribosome.

**Figure 5:**
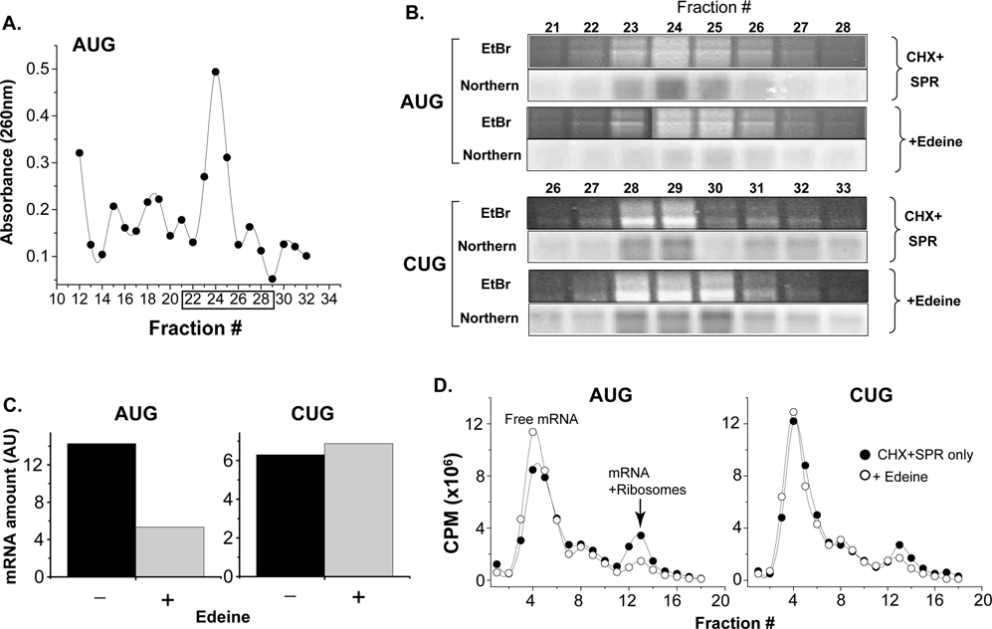
Edeine inhibits AUG- but not CUG-specific ribosomal initiation complexes. Ribosomes bound to the AUG[YL8] or CUG[YL8] mRNAs were fractionated on 10–40% sucrose gradients in the absence or presence of edeine (2 µM). (A) The ultraviolet light absorbance is shown for each fraction for reactions with the AUG[YL8] mRNA. (B) Total RNA from fractions 21–29 of AUG[YL8] and 26–33 of CUG[YL8] samples with or without edeine was fractionated on 1% formaldehyde gels. For each fraction, the ribosomal RNA from the large and small ribosomal subunits was visualized with ethidium bromide (EtBr) and the mRNA was detected by Northern blot. (C) mRNA amounts measured by pixel intensity of peak fractions of AUG versus CUG reactions in (B) is shown in arbitrary units (AU). (D) Sucrose gradient fractionation of AUG[YL8] and CUG[YL8] initiation complexes was carried out as in (A) except the mRNA was directly labeled with [α^35^S]-CTP and radioactivity (CPM, counts per minute) in each fraction was determined by liquid scintillation. Results are representative of three independent experiments.

Next, we analyzed the reaction mixtures by sucrose-gradient fractionation to confirm that the initiation complexes contained both 40S and 60S ribosomal subunits. Fractions were collected after ultracentrifugation of the initiation complexes layered onto 10–40% sucrose gradients. The absorbance of the individual fractions obtained with AUG[YL8] or CUG[YL8] mRNAs, shown in a representative experiment, revealed several distinct peaks with a maxima in fractions 21–26 for AUG[YL8] (**Fig. 5A**). We extracted RNA from these as well as surrounding fractions and analyzed the material on denaturing RNA gels (**Fig. 5B**). Bands corresponding to both 18S and 28S RNA were observed in an ethidium bromide-stained gel, with fractions 23–25 containing the highest amounts. The same fractions also contained the largest amount of mRNA when analyzed by a Northern blot (see methods for details). Thus, fractions 23–25 representing the predominant RNA absorbance contained the 40S and 60S ribosomal subunits as well as mRNA. However, in the presence of edeine the intensity of the AUG[YL8] mRNA band in the corresponding fractions was markedly reduced (**Fig. 5B,C**). Thus, edeine disrupted interactions between initiation complexes and the AUG mRNA. In contrast, when edeine was included in the reaction mixtures with the CUG[YL8] mRNA, there was little, if any, change in the fractions containing the mRNA (**Fig. 5B,C**).

To further confirm the differences in edeine sensitivity between ribosomes bound to either the AUG or CUG start codons, we used mRNA labeled directly with [α^35^S]-CTP (**Fig. 5D**, left panel). After fractionation of sucrose-density gradients, labeled AUG[YL8] mRNA was found in dense fractions (fractions 12–14) representing initiation complexes while a large amount of the total mRNA was free of ribosomes and present in the lighter fractions (fractions 3–6). In the presence of edeine, the initiation complex peak (mRNA+ribosomes) was reduced and there was a concomitant increase in the amount of free mRNA. In contrast, only a small difference in the initiation complex and free mRNA peaks was observed with CUG[YL8] mRNA (**Fig. 5D**, right panel). Thus, the assembly of AUG versus CUG initiation complexes can be distinguished by their sensitivity to edeine. Furthermore, this result shows that the difference in codon recognition is mediated through interactions within the decoding center of the 40S subunit.

### CUG initiation is resistant to small molecule inhibitors

**Figure 6:**
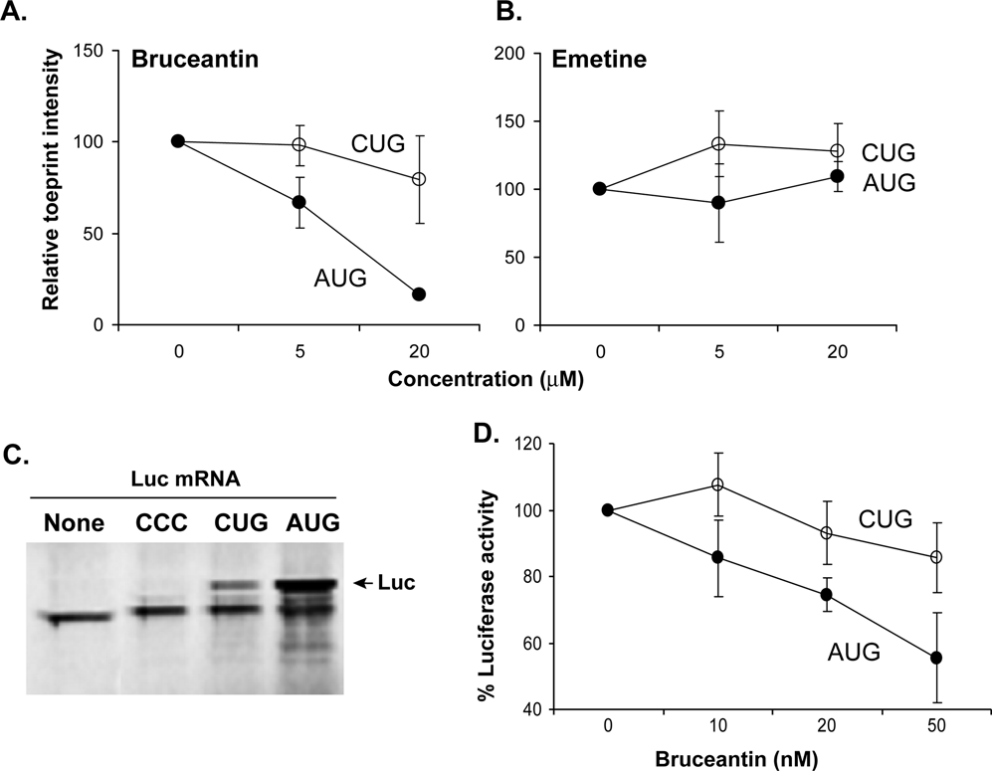
The toeprint and translational activity of CUG initiation codon is resistant to bruceantin. (A) The translation inhibitor bruceantin inhibits AUG toeprints, but not CUG toeprints in a dose-dependent manner. Bruceantin was preincubated with translational extract prior to adding the mRNA with the indicated AUG or CUG initiation codons. Relative toeprint intensity (% of untreated sample) represent three independent experiments (mean+/−standard error). (B) The AUG as well as CUG toeprints are insensitive to emetine, an elongation inhibitor. The data are representative of three independent experiments. (C) [^35^S]-Methionine labeled translated products of firefly luciferase (Luc) mRNAs with AUG, CUG, and CCC initiation codons *in vitro*. The translation products were resolved on 4–10% SDS-PAGE gel. Position of the 61 kD luc product is indicated by an arrow. Data are representative of three independent experiments. (D) Translation of AUG-Luc, but not CUG-Luc is inhibited by bruceantin in a dose-dependent manner. Indicated concentrations of bruceantin were added to the translation mix prior to the addition of mRNA. The luciferase activity was determined using a luminometer. Luciferase activity (% of untreated sample) is from two independent experiments (mean+/−standard error).

Although edeine distinguished the initiation complexes assembled at the AUG versus CUG codons, its various other side-effects preclude its use in living cells (data not shown). Therefore, we used the toeprinting assay to test a panel of translation inhibitors that distinguish conventional versus IRES mediated translation [27], [28]. Among the several compounds tested (data not shown), bruceantin, an irreversible inhibitor of initiation [28] which binds the large ribosomal subunit [29], was similar to edeine because it inhibited the AUG but not the CUG toeprint (**Fig. 6A** **and** **Fig. S1**). In contrast, and as a negative control, neither the AUG nor the CUG toeprint was affected by emetine, a potent elongation inhibitor (**Fig. 6B**). To directly assess the effect of bruceantin on protein translation *in vitro*, we generated luciferase (Luc) constructs with AUG, CUG, and CCC initiation codons and translated these mRNAs in rabbit reticulocyte extract (**Fig. 6C**). Measurement of the translated material showed that the amount of CUG-initiated Luc was approximately ∼10% of AUG-initiated Luc while the CCC codon did not support detectable translation. Notably, low nanomolar concentrations of bruceantin inhibited the translation of AUG-Luc more strongly than that of CUG-Luc (**Fig. 6D**). Thus, translation of AUG versus CUG-initiation codons *in vitro* could be distinguished by both edeine and bruceantin.

### Bruceantin distinguishes the presentation of AUG- versus CUG-initiated peptides

**Figure 7:**
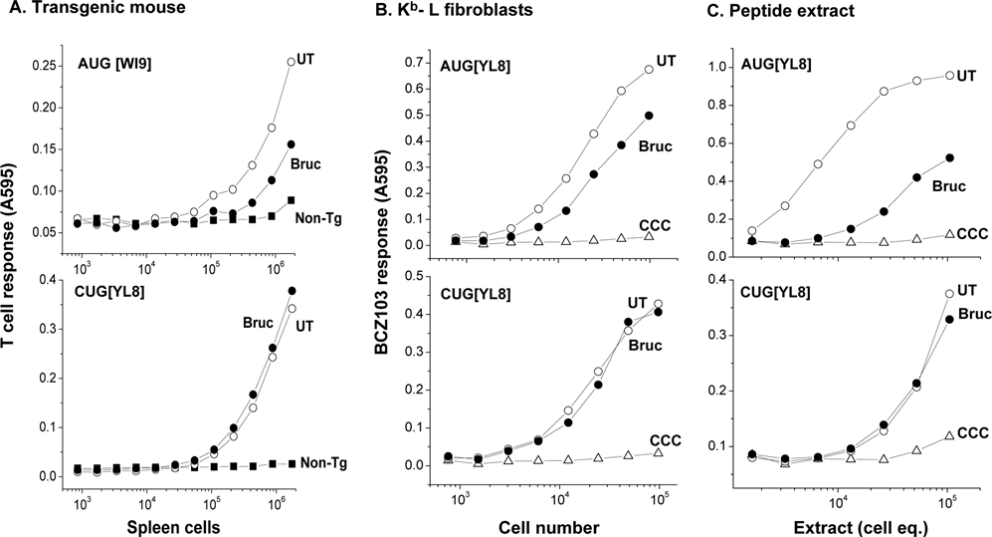
Generation of pMHC I complex derived from the AUG but not CUG mRNA is inhibited by bruceantin. (A) Primary cells from a transgenic mouse are sensitive to bruceantin inhibition from AUG but not CUG start codons. The transgene encodes two peptides: WI9 initiated with an AUG and LYL9 initiated with CUG located in the 3′ untranslated region directly downstream from WI9. Spleen cells were washed with mild-acid and allowed to recover for 3 hours in medium+DMSO or in the presence of 25 nM bruceantin. Peptide translation is measured with the 11p9Z and BCZ103 hybridomas for AUG and CUG initiation, respectively. (B) The K^b^-L cells were transfected with mRNAs encoding AUG[YL8] or CUG[YL8], and CCC[YL8] as a negative control. After three hours to allow mRNA entry and expression, the cells were incubated with 100 nM bruceantin for another three hours. The indicated numbers of cells were then used as antigen presenting cells for the BCZ103 hybridoma specific for K^b^-bound LYL8 or the MYL8 peptides. (C) Antigenic peptides in extracts of mRNA transfected cells in the absence or presence of bruceantin. After transfection, K^b^-L cells were acid-washed and allowed to recover for 3 hours in medium+DMSO or in the presence of 100 nM bruceantin. Peptides were extracted from the cells by homogenizing in 10% acetic acid, dried and antigenic activity measured with the BCZ103 hybridoma and K^b^-L cells as APC. The data are representative from four independent experiments.

In contrast to edeine, bruceantin was relatively non-toxic to tissue culture and primary cells (data not shown), making it possible to assess the effect of bruceantin on pMHC I translated via AUG versus CUG codons in living cells. We first used spleen cells from a mouse with a transgene encoding a conventional AUG-initiated AUG[WI9] and cryptic CUG[YL8] peptides [9]. The cells were first washed with mild acid to remove pre-existing surface pMHC I complexes and incubated with bruceantin during a 3 h recovery. As little as 10 nM bruceantin inhibited peptide supply as judged by expression of surface K^b^ MHC I molecules (data not shown). The spleen cells were also co-cultured with the WI9/D^b^ specific 11p9Z hybridoma [9], or the LYL8/K^b^ specific BCZ103 T cell hybridoma. The presentation of WI9 peptide encoded in the conventional AUG initiation context, despite being expressed at approximately one hundred fold higher level [9], was inhibited in the presence of bruceantin while that of CUG-initiated LYL8 peptide was unaffected (**Fig. 7A**). Thus, bruceantin differentially affected the expression of AUG versus CUG translation in primary spleen cells.

To rule out potential complications due to transcription of the transgene, we transfected K^b^ L-cells with mRNAs encoding the AUG[YL8] or the CUG[YL8] peptides. After allowing 3 hours for transfection, the cells were washed and treated with bruceantin for another 3 hours and then assayed for pMHC I expression with the MYL8 or LYL8/K^b^-specific BCZ103 T cell hybridoma (**Fig. 7B**). The response of the BCZ103 hybridoma to peptide derived from the potent AUG[YL8] mRNA precursor was reproducibly lower after bruceantin treatment while there was no detectable difference in the presentation of peptide derived from the CUG[YL8] mRNA. Because T cell responses to cells expressing minigene constructs can be saturated, we directly measured the translated material in peptides extracted from bruceantin treated cells. Again, the antigenic activity in extracts of AUG mRNA transfected cells was markedly reduced in the presence of bruceantin (**Fig. 7C**). In contrast, despite the weaker response to cells transfected with CUG mRNA (**Fig. 7C**), there was no detectable difference in the peptide activity in cell extracts. Thus, differential sensitivity to bruceantin indicates that CUG initiation also differs from canonical initiation at the peptidyl transferase center of the large ribosomal subunit.

Taken together with the *in vitro* toeprint and translation assays, these cellular assays show that initiation at the cryptic CUG codon can be distinguished from initiation at the canonical AUG codon. Because edeine and bruceantin exert their effects on the P site of the small and large ribosomal subunits respectively, distinct mechanisms are used to decode these initiation codons.

## Discussion

We show here that the translation mechanism for synthesizing cryptic peptides for presentation by MHC I is mediated through recognition of non-AUG initiation codons by ribosomes during the initiation step. Remarkably, the initiation mechanism for cryptic translation at CUG codon in these model antigens differs from that for conventional AUG codons by its sensitivity to inhibitors which affect interactions within the peptidyl transferase and decoding centers of the ribosomal P site. These findings provide insights into the mechanisms of cryptic translation at non-AUG start codons and suggest it could be used to regulate the peptide repertoire presented by MHC I molecules.

A large fraction of peptides presented by MHC I on the cell surface is derived from newly synthesized polypeptides [30], [31]. Beginning the antigen presentation pathway with newly synthesized material is particularly advantageous for immune surveillance of virus infected cells. The display of actively synthesized proteins as pMHC I allows CD8^+^ T cells to potentially detect intracellular viruses as soon as viral mRNAs are translated [32]. Furthermore, protein synthesis could be linked to generation of pMHC I in normal uninfected cells as well because the antigen presentation pathway is constitutively active in all cells.

The toeprinting method allows direct assessment of ribosomal binding to the mRNA obviating the complexities of various post-translational steps involved in generating pMHC I [33]. The toeprints, judged by the size of the reverse transcriptase product, reveal not only whether ribosomes are specifically bound to a particular sequence in the mRNA, but also the location of ribosomes at a single nucleotide resolution. With mRNAs encoding antigenic peptides, we determined that the toeprint at the CUG codon was identical in size to that of the AUG codon. In agreement with the low levels of the cryptic pMHC I expression, the amount of the CUG toeprint product was about 18–25% that of the AUG product (**Fig. 1**). Thus, the cryptic CUG codon in the mRNA was capable of not only engaging ribosomes but did so at the same location as the conventional AUG codon.

The exact location of the ribosome on the mRNA in the initiation step is critical because it determines the subsequent translational reading frame. The small 40S ribosomal subunit preloaded with initiator Met-tRNA_i_^Met^ scans in the 5′ to 3′ direction until an initiation codon, usually AUG, is found in a suitable nucleotide context [34], [35], [36]. The ribosome becomes functional when the 60S ribosomal subunit assembles with the 40S subunit bound to the initiation codon. At this juncture, Met-tRNA_i_^Met^ occupies the P site of the ribosome and the appropriately charged aminoacyl-tRNA specific for the next codon is recruited into the A site (**Fig. S1**). Remarkably, the CrPV IRES can direct initiation in the A site of the ribosome because toeprints were observed with GCU, a non-AUG codon encoding alanine, positioned in the A site, not the P site [15]. Thus, the CrPV IRES is similar to mRNAs used here because both use non-AUG codons and are decoded as non-methionine residues. Despite this similarity, ribosomes bound the CUG codon at the same location as the AUG codon. Therefore, translational initiation at the CUG codon does not involve a CrPV IRES-like activity and is similar to initiation at the conventional AUG codon. The similarity between conventional AUG and CUG recognizing ribosomes was further supported by the positive influence of the Kozak nucleotide context which enhanced both the toeprint intensity as well as the expression of the CUG derived pMHC I on the cell surface (**Fig. 2**).

Despite their similarities, the ribosomes recognizing the CUG initiation codon differed from those recognizing the AUG codon at both the large and small ribosomal subunit level. The MetRS inhibitor, methionine-sulfamide, reduced the binding of ribosomes at the AUG codon, but had relatively small effect on ribosomes binding the CUG codon (**Fig. 3**). This suggests that a significant fraction of 40S ribosomes which recognize CUG are either devoid of Met-tRNA_i_^Met^ or are preloaded with a different initiator-tRNA. The most obvious candidate for this function would be a leucyl-tRNA because the CUG start codons of antigenic peptides as well as human trypsinogen can be decoded with leucine [8], [9], [10], [37].

To further distinguish the ribosomes recognizing the CUG codon from conventional ribosomes we tested small molecule inhibitors in the toeprint assay. Edeine, a bacterial antibiotic isolated from *Bacillus brevis* [38], inhibits recognition of AUG start codons in mRNA by the small ribosomal subunit and profoundly inhibited the AUG toeprint in a dose-dependent manner **(** **Fig. 4**). Remarkably, the toeprint on the CUG codon was resistant to edeine. Structural studies have shown that edeine binds to the 40S ribosomal subunit and interferes with recognition of the AUG codon by Met-tRNA_i_^Met^ [25], [39]. Insensitivity of ribosomal recognition of CUG but not AUG to edeine is therefore, entirely consistent with the distinction between these ribosomes also seen with methionine-sulfamide.

Bruceantin, another small molecule like edeine, also inhibited the toeprints at the AUG, but not the CUG codon (**Fig. 6** **,** **7**). Bruceantin, isolated from the Ethiopian tree *Brucea antidysenterica* Mill. (Simaroubaceae) [40], is an irreversible inhibitor of translation initiation [28]. Bruceantin binds the large ribosomal complex at highly conserved nucleotides within the P site of the ribosome where initiator Met-tRNA_i_^Met^ binds [29]. Importantly, bruceantin inhibited AUG, but not CUG initiated translation of antigenic precursors in murine fibroblasts as well as normal spleen cells (**Fig. 7**). These observations show that the synthesis of polypeptides initiated by recognition of the CUG codon is mediated by initiation complexes which differ from those that initiate at the conventional AUG codon. Furthermore, because bruceantin binds nucleotides in the P site of the large ribosomal complex [29], and discriminates CUG from AUG initiation suggests that unique structural features distinguish these initiation complexes. This is also consistent with insensitivity of CUG binding ribosomes to edeine which further implicates the P site of the ribosome as the region responsible for distinguishing cryptic from canonical translation.

Interestingly, ribosomal heterogeneity has been invoked as a potential mechanism to explain complexities of biological phenomenon. For example, the ribosomal filter hypothesis of Mauro and Edelman suggests that interactions between mRNA sequences and heterogeneous ribosomes exerts a level of translational control during differentiation and development [41]. More recently, the functional analysis of duplicated genes of yeast ribosomal proteins by Silver and colleagues has revealed non-redundant roles in mRNA localization and translation [42]. Finally, and pertinent to antigen presentation, Yewdell and Nicchitta have argued in favor of an “immunoribosome”, dedicated to the efficient supply of polypeptides for presentation by MHC I [18]. The structural differences between all these subsets of ribosomes in eukaryotic models, as well as the mammalian ribosomes capable of distinguishing the AUG and CUG initiation codons described here remain unknown. Given the differential sensitivity of ribosomes in this system to edeine and bruceantin which directly bind ribosomal RNA [25], it is possible that they could be distinguished by the structural features of a unique initiator tRNA and/or ribosomal RNAs. The recent discovery of ribosomes containing mutant RNA which fail to translate IRES-containing mRNAs in X-linked dyskeratosis congenita is also consistent with this notion [43].

In conclusion, we have shown that initiation at the CUG codon for generating cryptic pMHC I can differ from initiation at the conventional AUG codon. While CUG initiation shares some similarities with AUG initiation, it diverges in its requirements and sensitivity to inhibitors which act within the initiation P site of the ribosome. Such cryptic initiation events could provide an important source of peptides for antigen presentation during viral infection or cellular stress when conventional translation mechanisms are subverted [44].

## Methods

### Experimental Procedures

#### Plasmid constructs

The cDNA constructs used for toeprinting and transfections were based upon those used earlier in functional antigen presentation assays [10]. The “Excellent Kozak” CTG[YL8] in the pcDNA1 vector (Invitrogen, Carlsbad, CA, U.S.A.): 5′- TGTGTAGTCGACCCTGACCTTCAACTACCGGAATCTCTAG-3′. The ATG[YL8] and CCC[YL8] constructs were identical in length and sequence to CTG[YL8] using the Sal I/Xba I sites from Excellent Kozak CTG[YL8] above; ATG[YL8]: 5′-GTCGACCATGACCTTCAACTACCGGAATCTCTAGA-3′ and CCC[YL8]: 5′-GTCGACCCCCACCTTCAACTACCGGAATCTCTAGA-3′. Firefly luciferase constructs (Luc) used for *in vitro* translation were prepared from Promega's T7 Luciferase Control Plasmid (Promega, Madison, WI, U.S.A.) by site-directed mutagenesis (Stratagene, La Jolla, CA, U.S.A.) of the ATG start codon as well as the upstream sequence to an ‘Excellent Kozak’ sequence: 5′-TCGACCCCC-3′, 5′-TCGACCATG-3′, and 5′-TCGACCCTG-3′, respectively. Cricket Paralysis Virus IRES-Firefly luciferase was in the pGEM-3 vector (CrPV-Luc) and was a kind gift from Peter Sarnow (Stanford University).

#### Synthesis of mRNAs

Plasmid DNA was linearized with Hpa I for pcDNA1 plasmids, Afe I for Luc plasmids, and Nae I for CrPV-Luc were used as templates for transcription by T7 RNA polymerase (RiboMAX Large Scale mRNA production system-T7; Promega or mMessage mMachine T7; Ambion, Austin, TX, U.S.A.) to yield CCC-, AUG-, CUG[YL8], CCC-, AUG-, CUG-Luc, and CrPV-Luc mRNAs, respectively. Transcription reactions contained m^7^GTP cap analog (Promega or Ambion) to yield naturally capped mRNAs. The Poly(A) Tailing Kit (Ambion) was used to add poly(A) tails onto mRNAs for cell transfections. Radiolabeled mRNA was prepared by including [α^35^S]-CTP (1250 Ci/mmole; Perkin Elmer, Waltham, Massachusetts, U.S.A.) in the transcription reaction.

#### Primer extension ‘toeprinting’ assay of initiation complexes

The 5′-end of the DNA oligonucleotide 5′-GTCACACCACAGAAGTAAGG-3′ used as the reverse primer was labeled with T4 polynucleotide kinase and [γ-^32^P]ATP (3000 Ci/mmol; Perkin Elmer). This primer is complementary to the mRNA at position +76 from the AUG/CUG start codons in the pcDNA1 vector. For toeprinting with CrPV-Luc, we used the oligonucleotide 5′-GCCTTATGCAGTTGCTCTCC-3′ which is complementary to the mRNA at position +86 from the GCU initiation codon. For toeprinting with emetine (Sigma) and bruceantin (obtained from the NCI/DTP Open Chemical Repository http://dtp.nci.nih.gov; NSC165563), the non-radioactive primer 5′-Alexa750 -GTCACACCACAGAAGTAAGG-3′ (Invitrogen) was employed. m^7^GTP cap analog was obtained from Promega and GMP-PNP was obtained from Sigma. Methionine- and phenylalanine-sulfamide were synthesized according to previously published methods [45].

The ribosome binding reactions utilized micrococcal nuclease-treated rabbit reticulocyte lysate (Flexi-rabbit from Promega). Reaction mixtures were assembled on ice in a total volume of 30 µL containing 50% (v/v) reticulocyte lysate, 500 µg/mL cyclohexmide, 200 µM sparsomycin, 2 mM DTT, 100 mM KCl, 0.5 mM MgOAc_2_, and any additional test compounds (e.g. edeine), and pre-incubated at 30°C for 5 min to allow the drugs to interact with the translational machinery. Template mRNA (0.5 µg/reaction) and 20 µM amino acids were added to allow initiation complex assembly at 30°C for 10 min.

The reverse transcriptase reaction was carried out in a total volume of 45 µL containing the entire ribosome binding reaction, 50 mM Tris-HCl (pH 8.3), 50 mM KCl, 10 mM MgCl_2_, 0.5 mM spermidine, 10 mM DTT, 500 µM of each dNTPs, 3.1 pmol ^32^P-primer (or 3.1 pmol Alexa750-primer for bruceantin (NCI) and emetine (Sigma)), and 10 U Avian Myeloblastosis Virus Reverse Transcriptase (RT, Promega). Reactions were incubated at 30°C for 35 min. Primer extension products were extracted by adding an equal volume pf phenol∶CHCl_3_ followed by precipitation with 1/10 vol. 3 M NaOAc (pH 5.2) plus 2.5 vol. ethanol. cDNAs were mixed with 40% formamide, 8 mM EDTA and heated to 95°C for 5 min before layering onto 8% polyacrylamide sequencing gel. For reference, RNA sequencing ladders were generated by primer extension with dideoxynucleotides using AMV. Dried gels were exposed on a PhosphoImager screen and analyzed using a Storm PhosphoImager (Molecular Dynamics). Gels loaded with Alexa750-cDNAs were visualized using an Odyssey Infrared Imaging System (LI-COR Biosciences, Lincoln, Nebraska U.S.A).

#### Sucrose gradient fractionation and Northern blot analysis of initiation complexes

Reactions for sucrose gradient fractionation were the same as for toeprinting except they were scaled up to 100 µL and contained 5 µg mRNA. After initiation complex assembly at 30°C for 10 min, reactions were diluted with 100 µL of 2× sample dilution buffer for a final concentration of 20 mM HEPES-KOH (pH 7.4), 100 mM KCl, 6 mM MgCl_2_, 2 mM DTT, 500 µg/mL cycloheximide and brought to 4°C on ice for 5 min prior to centrifugation using a SW-41 rotor (Beckman) for 2.5 h (39,000 rpm) at 4°C in 10–40% sucrose gradients containing 20 mM HEPES-KOH (pH 7.4), 100 mM KCl, 6 mM MgCl_2_, 2 mM DTT, 100 µg/mL cycloheximide. Gradients were manually fractionated (0.2 mL fractions: 32 fractions/gradient) and a portion was used for absorbance determination at 260 nm. RNA was extracted from the remaining portion (∼180 µL) of each fraction by addition of 200 µL of guanidine thiocyanate buffer (4 M guanidine thiocyanate, 25 mM sodium citrate, 0.5% N-lauryl sarcosine, 5 mM EDTA, and 0.1 β-mercaptoethanol) and 125 µL of saturated phenol. The samples were periodically rotated at RT for 15 min, followed by addition of 100 µL of CHCl_3_ and the samples were mixed and centrifuged at 5,000×g for 10 min at 4°C. RNA was precipitated with 1/10 vol. 3 M NaOAc (pH 5.2) plus 2 vol. ethanol, re-suspended in nuclease-free water and combined with RNA loading buffer (32 mM MOPS (pH 7.0), 1.6 mM EDTA (pH 8), 0.54% formaldehyde, 4% glycerol, 6.2% formamide, 10 µg/mL ethidium bromide), heated to 65°C for 10 min, and loaded onto a 1% formaldehyde gel (Ambion). RNA was transferred to a Hybond-XL membrane (GE Healthcare Life Sciences, Piscataway, NJ, U.S.A.) and probed with cDNA prepared from priming CUG[YL8] mRNA with the oligonucleotide 5′-Alexa750 -GTCACACCACAGAAGTAAGG-3′ (Invitrogen) in 0.25 M sodium phosphate buffer (pH 7.2), 1 mM EDTA, 1% BSA, 7% SDS, containing 100 µg/mL denatured salmon sperm DNA at 59°C. Blots were imaged on an Odyssey Infrared Imaging System. Sucrose gradient fractionation with radiolabeled mRNA was carried out as described above except 1.5×10^7^ CPM of body-labeled [α^35^S]-CTP mRNA was added per 100 µL reaction. Fractions were analyzed via liquid scintillation spectrometry.

#### In vitro translation

Translation reactions in a total volume of 50 µL containing 70% (v/v) RRL, 100 mM KCl, 0.5 mM MgOAc_2_, 20 µM amino acids, 40 U rRNasin (Promega) and test compound in no more than 1% DMSO were pre-incubated at 30°C for 5 min prior to the addition of AUG-, CUG-, and CCC-Luc mRNA and continued incubation for up to 1 h. A portion of the translation mix was allowed to equilibrate to RT and Luc activity was analyzed on a luminometer using Luciferase assay reagent (Promega). Incorporation of [^35^S]-Methionine into Luc protein from AUG-, CUG-and CCC-Luc mRNA was carried out as described above except each translation reaction contained 33 µCi of L-[^35^S]-Methionine (>1000 Ci/mmol; Perkin-Elmer) and 20 µM amino acids minus methionine (Promega). A portion of the translation reaction was combined with SDS gel-loading buffer (50 mM Tris-HCL (pH 6.8), 2% SDS, 0.1% bromophenol blue, 10% glycerol, 100 mM dithiotheitol), heated to 95°C for 5 min, and resolved using 4–10% SDS-PAGE.

#### mRNA transfections and T cell assays

L-cell fibroblasts expressing K^b^ and BCZ103 cells and their culture conditions have been described [46]. Cells were plated the night before at 5×10^5^ cells/well for 6-well plates or 4×10^6^ cells/dish for 10 cm dishes. The next day, poly(A) mRNA at 2 µg per well for 6-well plates or 10 µg for 10 cm dishes was used for 3 h transfections using TransMessenger Transfection Reagent (Qiagen, Valencia, CA, U.S.A.) according to the manufactur's recommendations. For bruceantin treatment, cells were rinsed with 1× PBS and fresh media containing bruceantin at the indicated dose was added for another 3 h. For acid wash experiments, transfected cells were treated with 0.131 M citric acid, 0.066 M NaH_2_PO_4_ pH 3.1 for 2 min, washed twice with 1× PBS, followed by addition of fresh media containing bruceantin with a 3 h incubation. Cell viabilty was was determined by subjecting treated or untreated cells to the CellTiter 96 Aqeuous Non-radioactive Cell Proliferation Assay (Promega) or by analysis of propidium iodide stained cells by flow cytometry. Extracts were prepared by resuspending APCs in 10% acetic acid and placed in boiling water for 10 min. Cell debris was removed by centrifugation at 13,000×g for 15 min and samples dried by vaccum centrifugation. Dried material was resupended in 1× PBS containing 25 µg/mL phenol red and the pH adjusted to 7 with 0.1 N NaOH and titrated in 96-well plates. APCs were added at 5×10^4^ cells/per well together with 1×10^5^ BCZ103 Lac Z-inducible T hybridoma [46]. The pMHC I induced accumulation of intracellular β-galactosidase in the hybridoma, was measured with the conversion of the substrate chlorophenol red-β-D-galactopyranoside with a 96-well plate reader at 595 nm and 655 nm as the reference wavelength [47]. Spleens from C57BL/6J (B6) mice were used to determine the effect of bruceantin on peptide supply. Transgenic mice have been described [9]. Spleen cells were treated with Puregene RBC Lysis Solution (Gentra Systems) to lyse red blood cells and subjected to mild acid wash as described above. After washing twice with 1× PBS, spleen cells were resuspened at 2×10^6^ cells/mL and treated with either DMSO or 25 nM bruceantin for 3 h. To determine the effet of bruceantin on peptide supply, spleen cells were stained with FITC α-mouse K^b^ Mab (AF6-88.5) or FITC mouse IgG2a, as an isotype control (BD Pharmingen). Stained cells were analyzed by flow cytometry with FlowJo data analysis software. For T cell assays, expression of the WI9/D^b^ or LYL8/K^b^ complexes was assessed by lacZ assays as described earlier [9].

## Supporting Information

Figure S1 Primer extension inhibition analysis ‘toeprinting’ on a model mRNA. Ribosomes in rabbit reticulocyte lysate were allowed to undertake the translation initiation step on natural globin mRNA but without translating the message. Initiation complexes containing ribosomes and other factors are stalled at the AUG start codon by the elongation inhibitors cycloheximide (CHX) and sparsomycin (SPR), which bind to the 60S ribosomal subunit and hence do not interfere with the initiation steps. The location of the ribosomal initiation complexes was identified by extending a [32P]-labeled complementary 3′ primer with reverse transcriptase (RT), up to the leading edge of the ribosome, 15–17 nucleotides downstream of the AUG codon (RT Stop). The resulting RT products were analyzed by gel-electrophoresis. The size of the fragments was measured at a single nucleotide resolution by comparison with sequencing reactions run on the same gel. Red boxes in the sequencing lanes indicate the location of the AUG codon and the toeprint at +15–17 nucleotides is boxed. The band at the top of the gel represents the full-length RT product up to the 5′-end of the mRNA. Unincorporated primer runs at the bottom of the gel. When edeine, an initiation inhibitor, is included in the toeprinting reaction along with cycloheximide (CHX) and sparsomycin (SPR), the toeprint is no longer observed and there is a concomitant increase in the intensity of the full-length cDNA band. Edeine and bruceantin bind the small and large ribosomal subunits, respectively.
(0.74 MB TIF)
