# Supplementary figures and images for: A Distinct Translation Initiation Mechanism Generates Cryptic Peptides for Immune Surveillance
Source: PLoS One. 2008 Oct 21;3(10):e3460. doi: 10.1371/journal.pone.0003460 (PMC2565129; doi:10.1371/journal.pone.0003460)

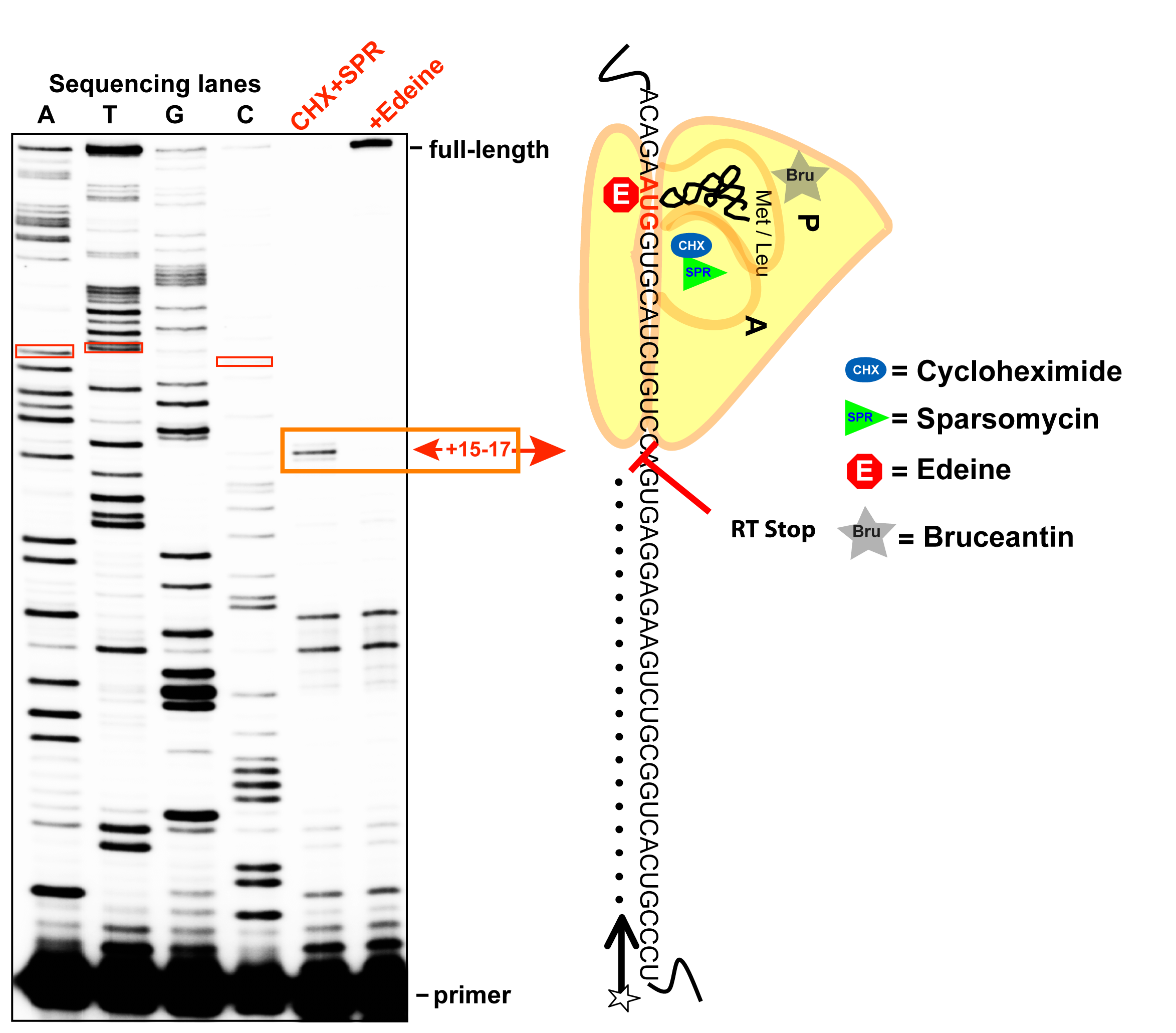

Supplement: Figure S1 — Primer extension inhibition analysis ‘toeprinting’ on a model mRNA. Ribosomes in rabbit reticulocyte lysate were allowed to undertake the translation initiation step on natural globin mRNA but without translating the message. Initiation complexes containing ribosomes and other factors are stalled at the AUG start codon by the elongation inhibitors cycloheximide (CHX) and sparsomycin (SPR), which bind to the 60S ribosomal subunit and hence do not interfere with the initiation steps. The location of the ribosomal initiation complexes was identified by extending a [32P]-labeled complementary 3′ primer with reverse transcriptase (RT), up to the leading edge of the ribosome, 15–17 nucleotides downstream of the AUG codon (RT Stop). The resulting RT products were analyzed by gel-electrophoresis. The size of the fragments was measured at a single nucleotide resolution by comparison with sequencing reactions run on the same gel. Red boxes in the sequencing lanes indicate the location of the AUG codon and the toeprint at +15–17 nucleotides is boxed. The band at the top of the gel represents the full-length RT product up to the 5′-end of the mRNA. Unincorporated primer runs at the bottom of the gel. When edeine, an initiation inhibitor, is included in the toeprinting reaction along with cycloheximide (CHX) and sparsomycin (SPR), the toeprint is no longer observed and there is a concomitant increase in the intensity of the full-length cDNA band. Edeine and bruceantin bind the small and large ribosomal subunits, respectively. (0.74 MB TIF) [file pone.0003460.s001.tif]
